# A Cause of Pulp Congestion

**Published:** 1904-11

**Authors:** M. C. Marshall


### A CAUSE OF PULP CONGESTION.

The subject of pulp-capping, like Banquo's ghost, will not down. The necessity for preserving this organ when exposed continually appeals to the progressive mind; and though failure after failure has been the history of this operation, with comparatively few exceptions, there are men who refused to be convinced that it cannot be successfully accomplished under favorable conditions.

So far as I am aware, the central thought has been to produce a substance to be placed in opposition to the pulp of such remedial and preservative character that this organ will continue its function in a healthy state, or else to so fix a concave disk of metal, the edges of which, resting on the dentin around the point of exposure, will protect it from actual contact with a foreign substance, thereby claiming to save it from destruction. So far as I know, a cause for hyperemia and death of all exposed pulps has been entirely overlooked, a condition that is not overcome or removed by any known method of capping. This cause is the irregular surface produced in the wall of dentin containing the pulp. This chamber is highly polished—lubricated to a greater or less degree by an exudate, which is indicative of nature's provision for protecting an easily irritated member. Hence, with an interruption of the continuity of this chamber, we find a condition that is directly the reverse of the smoothness originally provided. The edges of the break, even should they be smooth, will act as an irritant under the constant arterial pressure, and stasis in the pulp circulation must ensue. Just how far this might be retarded by a palliative covering I do not know, nor do I claim this broken wall to be the *only* cause for the almost constant death of exposed pulps; but I do claim that it is an ever present condition, which will in the vast majority of cases *in itself* produce a congestion that necessarily leads to the death of this organ. I am of the opinion that all our efforts at conservation of exposed pulps should recognize this cause of irritation, for I am convinced that no degree of success will or can attend this operation unless the continuity of this chamber is restored and perfectly smooth and compatible surface be left against which the pulsating pulp can rest and remain in a physiological state.—*Dr. M. C. Marshall, Era.*

